# “*e*LoriCorps Immersive Body Rating Scale”: Exploring the Assessment of Body Image Disturbances from Allocentric and Egocentric Perspectives

**DOI:** 10.3390/jcm9092926

**Published:** 2020-09-10

**Authors:** Johana Monthuy-Blanc, Stéphane Bouchard, Marilou Ouellet, Giulia Corno, Sylvain Iceta, Michel Rousseau

**Affiliations:** 1 GR2TCA-Loricorps-Groupe de Recherche Transdisciplinaire des Troubles du Comportement Alimentaire, Université du Québec à Trois-Rivières, 3351, Boulevard des Forges, Trois-Rivières, QC G8Z 4M3, Canada; marilou.ouellet@uqtr.ca (M.O.); michel.rousseau@uqtr.ca (M.R.); 2 Chaire de Recherche du Canada en Cyberpsychologie Clinique, Université du Québec en Outaouais, 283, Boul Alexandre-Taché, Gatineau, QC J8X 3X7, Canada; stephane.bouchard@uqo.ca (S.B.); giulia.corno@uqo.ca (G.C.); 3 Centre de Recherche du Centre Intégré de Santé et de Services Sociaux de l’Outaouais, Gatineau, 20 rue Pharand, Gatineau, QC J9Y 6H9, Canada; 4 Institut Universitaire de Cardiologie et de Pneumologie de Québec et École de Nutrition, Université Laval, 2325, Rue de l’Université, Québec, QC G1V 0A6, Canada; sylvain.iceta.1@ulaval.ca; 5 Centre Intégré et Spécialisé de l’Obésité de Lyon, Hospices Civils de Lyon, Pierre Bénite, France Université Claude Bernard Lyon 1, 69310 Lyon, France

**Keywords:** validation, body dissatisfaction, body distortion, state- and trait-BIDs, perceptual component, bodily feeling

## Abstract

The first objective of this study was to test the convergent and discriminant validity between the “*e*LoriCorps Immersive Body Rating Scale” and the traditional paper-based figure rating scale (FRS). The second objective was to explore the contribution of the egocentric virtual reality (VR) perspective of *e*LoriCorps to understanding body image disturbances (BIDs). The sample consisted of 53 female and 13 male adults. Body size dissatisfaction, body size distortion, perceived body size, and ideal body size were assessed. Overall, outcomes showed good agreement between allocentric perspectives as measured via VR and the FRS. The egocentric VR perspective produced different results compared to both the allocentric VR perspective and the FRS. This difference revealed discriminant validity and suggested that *e*Loricorps’ egocentric VR perspective might assess something different from the traditional conception of body dissatisfaction, which an allocentric VR perspective generally assesses. Finally, the egocentric VR perspective in assessing BIDs deserves to be studied more extensively to explore the possibility of finding two types of body image distortion: (a) an egocentric perceptual body distortion, referring to internal body sensation affected by intra-individual changes, and (b) an allocentric perceptual body distortion, referring to external body benchmarks constructed by inter-individual comparison occurring in a given cultural context.

## 1. Introduction

Body image concerns cause significant distress among males and females of all ages [1,2,3]. Likewise, body image disturbances (BIDs, mainly body dissatisfaction and body distortion) are a well-established risk factor for the development, maintenance, and relapse of eating disorders (EDs) [4,5]. Indeed, BIDs are common symptoms of anorexia nervosa, bulimia nervosa, and binge eating disorder [6,7,8].

The debate over a definition of body image is still open. Perhaps because of the complexity of this construct, researchers and clinicians have been struggling to find a final, universally accepted definition of body image. In this paper, body image is considered a multidimensional construct, indicating a personal mental representation of one’s physical appearance, encompassing body-related cognitions, emotions, behaviors, and perceptions [9,10,11]. Studies have suggested that body image is a dynamic construct closer to a state than a trait, influenced by everyday experiences, situations, or emotions [12,13,14,15]. BIDs can reveal themselves across cognitive-affective, behavioral, and perceptual dimensions of body image [9]. Body image distortion (perceptual dimension) and body image dissatisfaction (cognitive-affective dimension) are two of the most-studied manifestations of BIDs (e.g., [16]). Body image distortion has been defined as a disturbance in accurately estimating one’s own body size, whereas body image dissatisfaction refers to how much a person likes or dislikes the shape and/or size of their own body and whether they accept and value it [13]. Body image dissatisfaction has also been defined as the discrepancy between perceived and ideal body size [13].

Traditionally, body image dissatisfaction has been measured through self-report questionnaires, such as the Body Shape Questionnaire [17], Body Esteem Scale [18], Body Dissatisfaction Subscale of Eating Disorder Inventory 2 [19], Social and Physical Anxiety Scale [20], Body Image Avoidance Questionnaire [21], or by the discrepancy between the visual estimation of the perceived and ideal body using figure rating scales (FRS) and photograph distortion ([13,22,23,24]. In sum, body image dissatisfaction has usually been assessed using self-reported questionnaires and the FRS [13,25,26,27,28]. However, self-reported questionnaires are often associated with inconsistent and inconclusive results due to the heterogeneity of psychometric instruments, as underscored by Fisher et al. [29], as well as the lack of consensus concerning conceptual bases of body image disturbances (BIDs) [28]. The alternative—the paper-based FRS—can be criticized for its unrealistic representations of one’s own body—its lack of ecological validity due to the exclusive use of frontal views of bodies, and the incomplete representation of the full range of body sizes in the population, such as obesogenic body types [30].

In regard to the lack of ecological validity due to the 2D visual perspective, in the last two decades, virtual reality (VR) technology has increasingly been used to better understand the concept of body image and to assess and treat BIDs. The research team led by Riva conducted the first pioneering studies of the application of VR to assess and treat BIDs [31,32]. They developed a software package, the BIVRS (Body Image Virtual Reality Scale; [31,33]), with a 3D graphical interface representing nine male or nine female figures ranging from underweight to overweight. All figures were represented in an allocentric (i.e., third person) perspective, as in the classical paper-based FRS. This perspective corresponded to a non-immersive VR modality. Participants could choose the figures that best represented their mental representation of their actual and ideal body size. The discrepancy between perceived and ideal body size was adopted as a measure of body image dissatisfaction [31,33]. The BIVRS software was used by Riva and colleagues in several studies for assessing changes in BIDs after treatment [13].

In Spain, Perpiña and colleagues [34] designed and developed another VR software for the assessment and treatment of BIDs in EDs. Their VR application consisted of a 3D virtual human body, presented in an allocentric perspective, whose body parts could be modified by the participant using a slider bar. The results showed that this tool allowed patients to model and reflect mental representations of their actual and ideal body size [34]. However, no psychometric studies about these assessment tools have been published [13]. Following these pioneering works, a growing number of studies have used VR to attempt to assess BIDs by manipulating the perceived body size [13,35,36,37].

The human spatial experience involves the integration of information from two different perspectives: (a) egocentric (i.e., the first-person perspective of reference, as perceived through our own eyes) and (b) allocentric (i.e., the third-person perspective of reference, with the body being perceived as an object integrated into the surrounding physical environment) [38].The Allocentric Lock Theory posits that the spatial egocentric perspective of reference could have its source in somatoperceptions, which are representations of the actual state of the body and tactile stimuli from sensory inputs, whereas the spatial allocentric perspective of reference could derive from somatorepresentations, which shape the “memory of the body” [38,39,40]. Somatorepresentations include knowledge, beliefs, and attitudes related to one’s own body. This theory proposes that individuals with (or at high risk of developing) an ED may be “locked” to a negative allocentric memory of their own body. Specifically, it is possible that EDs patients are locked—by cognitive-affective BIDs—to a mental representation of their body that is no longer updated by perceptual inputs, even after drastic body shape and size changes [41].

A growing number of studies have recently explored the potential of VR technologies by presenting scenarios through allocentric perspectives (e.g., as if immersed users are looking at their image in a mirror) [42,43]. However, VR also makes it possible for participants to look at themselves from an egocentric perspective (e.g., as if they are inside the virtual bodies). In addition to the possibility of exploring allocentric and egocentric representations of the body, VR technology allows for simulating real-life situations related to body image concerns (e.g., being looked at by other people). It also provides confidential, flexible, and controlled environments where participants can be exposed to emotionally-challenging situations. In VR, people are more than merely rating figure drawings of various body sizes. Immersive technologies track people’s head and body movements, allowing the user to navigate around virtual bodies, move closer or farther from body parts, and assess realistic 3D representations of the human body perceived from multiple angles.

VR provides two main approaches for the assessment of BIDs: (a) VR body size modulation task and (b) VR body continuum task. In a VR body size modulation task, the user adjusts the size of each part of a virtual body to recreate their mental representation of their own body size. This approach allows the user to project their personal mental representation of the body onto the creation of a virtual body. Previous studies have shown that participants tend to underestimate their weight but not their body dimension in VR body size modulation tasks (for normal-weighted participants [44] or those suffering from anorexia nervosa [45]). Moreover, body size modulation has the advantage of allowing users to “carve” specific areas of the virtual body to fit their personal mental representation.

Conversely, VR body continuum tasks share similarities with the traditional paper-based FRS. Typically, the user immersed in VR is invited to rate or select from a line-up of several virtual bodies. This method allows the user to compare various virtual bodies, adding an element of social comparison that is often important in BIDs [46]. Using a VR body continuum task, Fisher et al. [29] compared ratings of virtual bodies to ratings of classical paper-based FRS by 31 anorexic adolescents. Ten virtual female bodies were displayed and evenly spaced in a circle around the user immersed in VR. The participant could observe each virtual body, numbered from 1 to 10, and illustrating a progressive increase in body mass index (BMI) from 10 to 30 kg/m^2^. Participants were asked to indicate which virtual bodies corresponded to their perceived and ideal body size. Time spent looking at each virtual body was also measured. The authors found no significant difference in average score ratings obtained through the paper-based and the VR-based FRS. Furthermore, the correlations between body dissatisfaction (calculated using the VR-based FRS) and measures of BIDs were significant (drive for thinness (Eating Disorder Inventory 2; EDI-2), 0.50; body dissatisfaction (EDI-2), 0.47; body dissatisfaction (Body Shape Questionnaire; BSQ), 0.41) but moderate. Although correlations between the VR-based body ratings and the paper-based FRS would have contributed to understanding the convergent validity of the immersive method and provided more nuanced interpretation than a lack of significant difference in average scores, they were not reported. Results from Fisher et al. [29] were a significant first step in validating the virtual body continuum task in the assessment of BIDs. However, in their VR-based FRS the authors developed and used virtual bodies presented only in an allocentric perspective. Therefore, they did not explore the potential uses of and differences between the allocentric and egocentric perspectives.

Corno and colleagues [42] used allocentric and egocentric perspectives to assess attitudinal and perceptual factors associated with BIDs through the immersion in a virtual environment in which users’ avatars could move their arms and bodies in real-time. Their procedure consisted in a VR body size estimation (which is a paradigm close to body size modulation task). Participants started with a virtual body of 20.5 kg/m^2^ in either an allocentric (i.e., on a virtual mirror facing the user) or an egocentric (i.e., a user looking down at the body) perspective. They changed the virtual body until it corresponded to their perceived body size. Although some of the 27 female participants displayed signs of dysfunctional weight-related attitudes based on the questionnaires, on average the sample was in the normal BMI range. Participants switched and chose from a sample of virtual bodies with BMIs spanning from 12.5 to 42.5 kg/m^2^. Cognitive-attitudinal measures of BIDs (i.e., body dissatisfaction, body uneasiness, and body image avoidance) were assessed with self-report questionnaires. In both the allocentric and egocentric perspectives, the authors found no significant difference between the estimations of virtual-body and actual BMI. However, the authors found that in the allocentric perspective, body dissatisfaction, body image avoidance, and body uneasiness were significantly related to BIDs. More specifically, the overestimation of body size was predicted by body image avoidance, and the underestimation of body size was predicted by body uneasiness. Body dissatisfaction was a predictor of both underestimation and overestimation. The above BIDs’ attitudinal components were not significantly related to perceptual BIDs in the egocentric perspective. The study supported the hypothesis that allocentric and egocentric perspectives could provide different information on BIDs. However, Corno et al. [42] did not include the classical paper-based FRS to interpret their results in the context of current knowledge on body image distortion and body dissatisfaction.

Two objectives—psychometric and explorative—characterized the present study. The first objective (O.1) was to test the convergent and discriminant validity between the traditional paper-based FRS and the VR-based FRS “*e*LoriCorps Immersive Body Rating Scale”. Specifically, convergent validity focused on the allocentric perspectives from the paper scale and VR-based FRS, as the perception should be the same when assessed with two different methods. Discriminant validity focused on the allocentric and egocentric perspectives within a similar method (VR-based FRS) and across different methods (paper vs. VR). It was expected that correlations between allocentric perspectives measured with different methods would be higher than between allocentric perspectives (both paper-based and VR-based) and egocentric VR perspective. The second objective (O.2) was to explore the contribution of the egocentric VR perspective of *e*LoriCorps to our understanding of BIDs. First, components of BIDs—body distortion and body dissatisfaction—in the allocentric VR perspective versus in the egocentric VR perspective were compared. Second, the relationships between components of BIDs in the egocentric VR perspective and psychometric measures of BIDs were explored.

## 2. Experimental Section

### 2.1. Sample

Participants were 53 women (78.9%) and 13 men, all Caucasian. Ages ranged from 18 to 52 years (M = 23.2 ± 5.3). The average weight and BMI of the participants were, respectively, 65.12 (±12.4) and 23.24 (±4.8). Among the participants, 91.2% were Canadian, and 8.8% were French. They were recruited at the Université du Québec à Trois-Rivières (Trois-Rivières, Québec, Canada), via brief classroom presentations and emails sent to university students, faculty, and staff. Inclusion criteria required that participants be francophone women or men aged 18 and over. One participant was excluded during the study due to cybersickness, yielding a sample size of n = 65 (Simulator Sickness Questionnaire’s mean score for the sample: 18.47 ± 2.8 (corresponding to a low level of cybersickness)).

### 2.2. Equipment and Material

**Figure 1:**
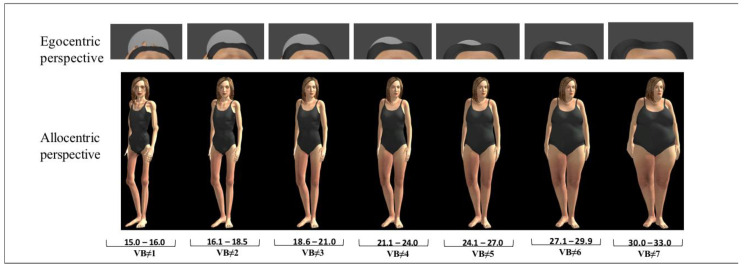
Allocentric and egocentric perspectives of *e*Loricorps in females. Note. VB: virtual body.

**Figure 2:**
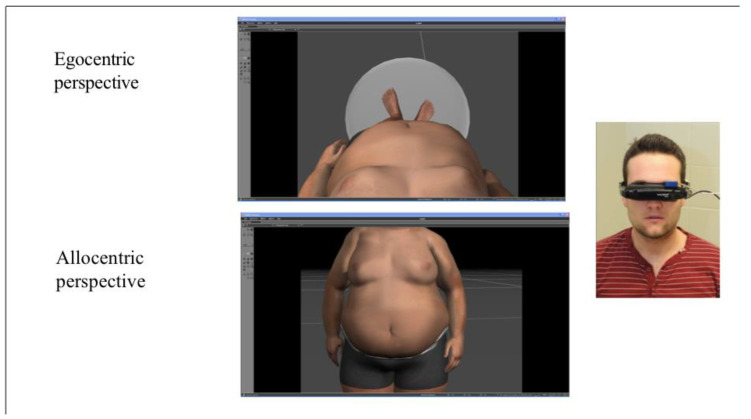
Allocentric and egocentric perspectives of *e*Loricorps in males.

*e*LoriCorps is a VR program running on an HP wx4600 PC computer (3 GHz, 3.48 GB RAM, ASUS GeForce 8800GTX graphics card; Hewlett-Packard, Montréal, QC, Canada), combined with Vuzix VR920 HMD (Vuzix, Rochester, New York, NY, USA), an Intersense Cube3 motion tracker (InterSense LLC, Billerica, MA, USA), and a hand-controlled joystick from a wii RVL-003 (Nintendo Canada, Vancouver, BC, Canada). This program immersed participants in a virtual environment in which they saw seven virtual bodies matching their reported sex and ranging from underweight to overweight with a standardized increase in BMI (see Figure 1 and Figure 2).

The development of *e*LoriCorps was based on the initial validation of the paper-based version of the Body Rating Scale [26], which consisted of seven female or seven male body figures drawn in black and white, ranging from 1 to 7 with BMI increasing from 15.00 to 33 kg/m². Each virtual body of *e*LoriCorps was created by a graphic designer in a T-pose position with 3D Studio Max (Autodesk Canada, Montréal, Québec, Canada). Virtual body #4, with normal BMI, was the first to be created. Using the 2D measurement of the fourth FRS body figure (corresponding to a BMI of 21.75 kg/m^2^, in the middle of the range of World Health Organization normal BMI category), each part composing volume of the body (e.g., waist, chest, etc.) was developed to create virtual body #4. Then, shading and lighting were used to make body areas more realistic. Specifically, the female virtual body was wearing a grey two-piece swimsuit, and the male virtual body was wearing grey cycling shorts. Gardner et al. [25] suggested omitting facial and body features reflecting ethnicity in paper-based scales. Caucasian characteristics, such as light skin and brown eyes and hair, were chosen to accurately represent the context of the current sample. Using 3D Studio Max, virtual body #4 was altered to create three virtual bodies, which were successively thinner than virtual body #4 (with a decrease of 30% between each pair of virtual bodies), and three virtual bodies, which were successively bigger than virtual body #4 (with an increase of 30% between each pair of virtual bodies). Thus, seven virtual bodies were created in total from the thinnest to the biggest, arranged in a line-up facing the user rotated at an angle of 36 (as in the paper-based FRS) and with sufficient space between bodies to allow a user to walk around one body without virtually hitting another one. This was done for both the female and male bodies, creating two versions of the scale. In the end, seven 3D standardized female and male virtual bodies were created to match the number of characters illustrated in the paper-based FRS used in this study.

This study included the use of three virtual environments: a neutral virtual environment and two experimental virtual environments (in a randomly counterbalanced sequence). The neutral environment (Step 1) allowed the users to acquire the navigation skills required to move around virtual bodies and become accustomed to immersions in VR without actually being exposed to the experimental stimuli. When immersed in this virtual environment, users were located on the street in a virtual city and were told to use the hand-held controller to cross the street and enter a pub. The users were asked to approach the bartender who was behind the counter, making sure not to hit the bar; look him in the eyes; then move to the far end of the pub, again while paying attention to avoid collisions with furniture. Upon reaching the far end of the pub, users closed their eyes, and the experimenter launched the next VR immersion (Step 2). Users were invited to open their eyes to see, in an allocentric perspective, a line-up of seven virtual bodies of increasing BMIs, from 15 to 33 kg/m². All seven virtual bodies were visible in the user’s field of vision, with virtual body #4 located directly in front of the user. In this condition, the users could move into the environment with the controller. The experimenter explained to the users that they must use the controller to observe and walk around each virtual body in sequence, beginning with virtual body #1 and ending with virtual body #7. The exploration of each virtual body lasted roughly 40 to 60 s. When the users had completed the exploration task on virtual body #7, the experimenter asked them to walk to face the virtual body that best represented their own body (i.e., perceived body size), then to walk to the one they wanted to look like (i.e., ideal body size), and then to close their eyes. While users had their eyes closed, the experimenter launched the last virtual environment (Step 3). Before opening their eyes, users were asked to look down and keep their heads down, as if looking at their feet. When the immersion began, users were immersed in virtual body #4 in an egocentric perspective and were told that they could experience the virtual body as if it were their own, looking from the chest down. In this condition, the user could move their head along three degrees of freedom (yaw, pitch, and roll). Then, the experimenter explained that users would experience each virtual body, from the thinnest to the biggest, beginning with virtual body #1. The experimenter used keyboard strokes to control the transitions from one virtual body to another, using progressive fade-ins and fade-outs. During the exploration, users were immersed in and experienced each virtual body for 40 to 60 s. When users said that they had finished experiencing and observing virtual body #7, they were brought back into virtual body #4 and asked to guide the experimenter in transitioning to the virtual body they estimated as best representing their perceived body size. Transitions from one body size to the next were experienced in the egocentric point of view until the desired target was reached by the users. The same procedure was repeated for the ideal body size, starting again from virtual body #4.

### 2.3. Assessment Measures

The sociodemographic questionnaire included height, weight, nationality, sex, and date of birth. These variables were assessed to have a picture of the sample’s participants.

The FRS [26] is a paper-based questionnaire consisting of seven figure silhouettes that increase in size from thinnest to biggest, numbered from 1 to 7, respectively. In our study, this tool was used as the paper-based FRS. Participants were asked to circle their perceived body size and their ideal body size. From these answers, we calculated body dissatisfaction and body distortion scores.

The French short version of the Eating Disorder Inventory (EDI-A; [47]) is a 24-item multidimensional self-report questionnaire that assesses symptoms of eating disorders. The questionnaire comprises eight subscales and is based on a Likert scale from 0 “not at all” to 5 “extremely.” In this study, only subscales that were relevant to our present objectives were kept (i.e., body dissatisfaction, drive for thinness, and bulimia) for a total of 10 items. The total for each subscale was reported. In our sample, Cronbach’s alpha was 0.887, showing good internal consistency.

The Social Physical Anxiety Scale (SPAS-12 [20], validated in French by Maïano et al. [48]) is a 12-item self-report scale developed to assess the degree to which people become anxious when others observe or evaluate their physiques. The questionnaire is based on a Likert scale from 1 “not at all” to 5 “extremely”. In our sample, Cronbach’s alpha was 0.795, indicating internal consistency.

The French version of the Simulator Sickness Questionnaire [49] measures cybersickness, the presence of physiological discomfort during VR immersion. The 16-item questionnaire uses a Likert scale from 1 “not at all” to 4 “severely”. In our sample, Cronbach’s alpha was 0.718, indicating good internal consistency.

### 2.4. Procedure

The study protocol was approved beforehand by the ethics committees of the two universities involved (Université du Québec à Trois-Rivières (UQTR) and Université du Québec en Outaouais, CER-11-172-06.01). Participants were recruited exclusively from UQTR via a short description of the study displayed on the university home page, sent by electronic mail to all students, and presented during classroom visits. During a meeting at the UQTR research lab, participants first answered sociodemographic questions asked by the research assistant. Their height and weight were measured without shoes. Then, they answered paper-based questionnaires (EDI, SPAS) and the classical paper-based FRS independently. Next, *e*LoriCorps was administered by the research assistant. Before the test administration, the research assistant explained the procedure to participants and gave them bottled water. Participants were informed that some cybersickness could occur and were encouraged to mention if it happened. After immersion in the three virtual environments (neutral and both experimental environments), participants were invited to document their immersive experience. Finally, the Simulator Sickness Questionnaire was administered to all participants. After the experiment, participants were debriefed by a clinician and discussed their impressions and feelings towards the immersions.

### 2.5. Statistical Analysis

The participants with missing data (n = 2) were excluded from the analysis. To document the potential impact of gender on the results, all statistical analyses were also performed separately for females and males. The results did not differ when analyzed separately for each sex (i.e., significant differences remained significant, and non-significant differences remained non-significant). Therefore, to maximize statistical power, results for the aggregated sample were reported (results analyzed by sex are available upon request). Parametric variables were represented as mean ± standard deviation (SD). Statistical analysis was performed using Stata 16.1 software. In the paper-based FRS and *e*LoriCorps conditions, score amplitude indicated the severity of BIDs, with a score of zero indicating no BIDs. Body distortion corresponded to the actual BMI of the participant minus the BMI of the perceived body size (see Table 1). A positive score indicated that the participant perceived their body as thinner than their actual BMI, while a negative score meant that the participant perceived their body as bigger than their actual BMI. A score of zero indicated that the perceived body size of the participant corresponded to the actual BMI (i.e., no body distortion existed). Body dissatisfaction corresponded to the perceived body size minus the ideal body size. A positive score indicated that the participant desired a thinner body than their perceived body size, and a negative score indicated that the participant desired a bigger body than their perceived body size. A score of zero indicated that the ideal body size of the participant corresponded to their perceived body size (i.e., no body dissatisfaction existed). Body distortion and body dissatisfaction scores ranged from 0 to 6. Body dissatisfaction and body distortion were calculated for allocentric and egocentric perspectives (O.1). Given the ordinal nature of the scores, the Spearman correlation was used to measure the convergent and discriminant validity of the paper-based and VR-based scores (O.2). Comparisons between allocentric and egocentric VR scores were performed with paired student’s t-tests using Cohen’s D as the measure of effect size. Pearson correlation was performed to check the relationship between paper-based and VR-based body measurements and extraneous variables. Because multiple hypotheses were being tested in this study, a Bonferroni correction was applied to the alpha level, resulting in any *p*-value lower than 0.002 (0.05/22) to allow for a statistically significant result. For the BMI, two outliers were found in the data. Raw BMI was never used in the analysis for this paper. It was rather transformed into body score, a categorical variable, and used to compute the body distortion score. In doing so, any potential effect of the two outliers on the results was neutralized. Besides, the data were normally distributed, and removing the two highest BMI did not influence our results.

## 3. Results

Ratings of the BID scores and their components (actual and ideal body scores) are presented in Table 1. Results showed that the paper-based FRS presented the highest average for all scores, except the body distortion score and ideal body score. Specifically, body distortion showed the lowest average score compared to VR conditions. The egocentric VR condition results showed the highest variation between respondents for all reported scores. Unlike in the allocentric VR condition, some respondents in egocentric selected the most extreme body forms in the continuum (i.e., #1 and 7). In the egocentric VR condition, body dissatisfaction scores showed positive average values, which meant that participants generally perceived themselves as bigger than their ideal body size. In the egocentric VR perspective, results showed that participants essentially saw themselves as the same size as their ideal body size, as the average score was negative but very close to zero (considering that a value of one point separated adjacent virtual bodies). For the body distortion score, positive average values were found, which meant that, on average, participants perceived themselves as slightly smaller than their actual body size (reflected by their actual BMI).

Regarding the first objective (O.1), Table 2 shows Spearman correlations between ratings using the classical paper-based FRS mode and the allocentric and egocentric VR conditions. For most of the scores, correlations between paper-based and allocentric VR were higher than between paper-based and egocentric VR as well as between VR administration modes. This pattern was expected because the paper-based FRS and allocentric VR condition utilized a similar perspective with different methods. Conversely, egocentric VR used a different perspective than allocentric VR and a different perspective and method than paper-based FRS. For ideal body size scores, no significant correlation between different administration modes was found. Besides, a significant correlation between body distortion scores across the different administration modes was found, with a higher effect size than for the body dissatisfaction scores.

**Figure 3:**
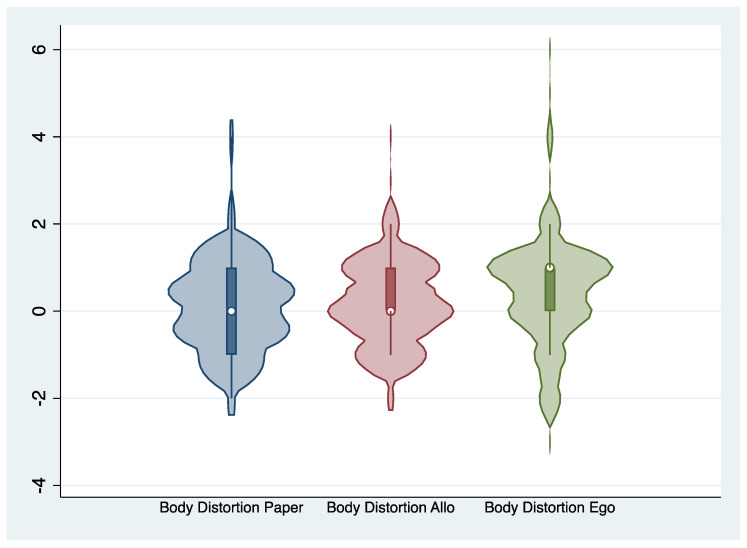
Violin plot of the distribution of body distortion scores.

**Figure 4:**
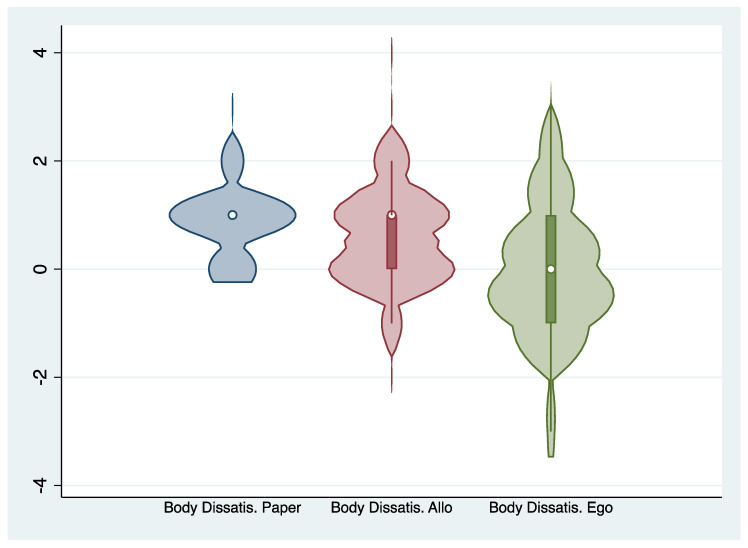
Violin plot of the distribution of body dissatisfaction scores.

Regarding the second objective (O.2), paired t-tests were conducted to compare mean scores of body distortion and body dissatisfaction for the two VR conditions. For body distortion, the score was higher in the egocentric perspective than in the allocentric perspective, but the difference was not statistically significant (t = −2.02, df = 64, *p* = 0.047), and the Cohen’s D was −0.25, corresponding to a small effect size. For body dissatisfaction, the average score was higher in the allocentric VR perspective, and the difference between those two averages was statistically significant (t = 3.94, df = 64, *p* < 0). Cohen’s D for this comparison was 0.49, corresponding to a medium effect size. Body distortion and body dissatisfaction scores regarding Allo or Ego VR are represented in Figure 3 and Figure 4. Table 3 presents the correlation between body dissatisfaction and body distortion scores and external variables. The only statistically significant correlation, after Bonferroni’s correction, was between allocentric VR and SPAS-12 score. This relationship was relatively strong, with a correlation of 0.489. The correlation with the EDI-A body dissatisfaction subscale was lower than with the SPAS-12.

## 4. Discussion

The first objective (O.1) of this study was to test the convergent and discriminant validity between the traditional paper-based FRS and the VR-based FRS “*e*LoriCorps Immersive Body Rating Scale”. As expected, overall outcomes showed convergent validity between allocentric perspectives in the traditional paper-based FRS and *e*LoriCorps in all BID dimensions, except the ideal body size. Thus, convergent validity existed between two different modes of administration (i.e., classic paper-based FRS vs. allocentric VR perspective of *e*LoriCorps) to assess BIDs. The egocentric VR perspective produced different results compared to the traditional paper-based FRS and the allocentric VR perspective in *e*LoriCorps. The second objective (O.2) was to explore the contribution of the egocentric VR perspective in *e*LoriCorps to understanding BIDs. Overall results tended to show that the egocentric VR perspective addressed something different from the traditional conception of body dissatisfaction, generally evaluated in an allocentric VR perspective. Finally, the egocentric VR perspective on BIDs deserves to be studied more extensively to explore the possibility of finding two types of body distortion: (a) an egocentric perceptual body distortion, referring to internal body sensation affected by intra-individual changes (mood, physiological hunger, and satiety sensations, etc.), and (b) an allocentric cognitive body distortion, referring to external body benchmarks constructed by inter-individual comparison occurring in a (Western) cultural context. These results and interpretations are further discussed in the following paragraphs.

### 4.1. Convergent/Discriminant Validity (O.1)

For the allocentric perspective, the highest observed agreement was between the traditional paper-based FRS and *e*LoriCorps for perceived body size, body distortion, and body dissatisfaction, which reflected good agreement between the two administration modes to measure BIDs. Correlation patterns confirmed the convergent hypothesis (except for ideal body size). Correlations for ideal body size scores were rather low among all modes and conditions. Results were in line with those from Fisher et al. [29], who demonstrated that body distortion (i.e., body perception index (BPI), [29]) did not differ significantly between traditional paper-based FRS and their allocentric perspective using a VR system relatively similar to *e*LoriCorps for body distortion, body dissatisfaction, perceived body size, and ideal body size. Even if fairly good discriminant validity exists for the allocentric VR perspective, findings are more balanced for the egocentric perspective, especially regarding correlation with ideal body size and body dissatisfaction. More precisely, for ideal body size, it seems that the method has an important impact on the scores. However, lower variation within the egocentric perspective scores could explain those lower correlations. Indeed, the size of a correlation is directly influenced by the variance of the variables used to compute its value. In conclusion, in the case of the egocentric VR perspective, the smaller correlations with both allocentric perspectives (i.e., paper-based and VR) suggest that the egocentric modality may be tapping into something different from the allocentric perspectives.

### 4.2. Egocentric VR-based Perspective Exploration (O.2)

For body distortion, the difference was not statistically significant between the two perspectives. These results were in line with Corno et al.’s study [42]. In that study, a virtual body without a head or facial features was presented, and participants moved their hands up and down for 90 s. The fact that the egocentric immersive procedure differs from one study to another, but the outcomes are identical, reinforces the specific link between body distortion and egocentric perspectives and its future implication. Because body distortion is a central feature of EDs’ pathology [6], it is possible that this symptom is generalizable and measurable in both VR perspectives of *e*LoriCorps. The egocentric (vs. allocentric) perspective of *e*LoriCorps, as theorized by the Allocentric Lock Theory, could reflect an individual perception of their body size without being influenced by attitudinal-cognitive-affective variables [33]. The relationship between body distortion in an egocentric perspective and other constructs associated with BIDs differed according to the dimensions of body image. Thus, there was no significant correlation between body distortion in the egocentric perspective and eating disorders symptoms EDI (except drive for thinness), whereas a significant correlation was found with social physical anxiety. It seems that body distortion in the egocentric perspective was not related to traditional cognitive-affective-attitudinal dimensions of BIDs, unlike with body distortion in the allocentric perspective [42]. Moreover, the relationship between body distortion and both egocentric and allocentric perspectives was comparable to Porras et al.’s conclusions [49] on the usefulness of VR embodiment-based techniques to induce changes to body anxiety in a non-clinical sample of college students.

To our knowledge, these were the first available results comparing body dissatisfaction between allocentric and egocentric VR perspectives with tools similar to paper-based figure rating scales. Of note, Corno et al.’s study [42] compared egocentric and allocentric perspectives in a body estimation task with a procedure that did not involve several virtual bodies. The difference in findings between egocentric and allocentric perspectives might suggest a role for social comparison factors. The comparison of BIDs between egocentric and allocentric VR perspectives showed less body dissatisfaction in the egocentric VR perspective of *e*LoriCorps. In other words, this revealed an interesting discriminant validity and suggested that the egocentric VR perspective might address something different from the traditional conception of body dissatisfaction, generally evaluated in the allocentric VR perspective. Moreover, no significant correlation was found between body dissatisfaction in the egocentric perspective and all symptom subscales of EDI, and, more surprisingly, with the body dissatisfaction subscale. This could be explained by EDI measuring body image dissatisfaction in differentiated ways (e.g., partial body shape, size, weight), while VR perspectives of *e*LoriCorps measured the overall body size dissatisfaction. Indeed, based on an adjusting and morphing virtual body task, Thaler et al. [44] found that on working with a virtual body, participants underestimated their weight but estimated their body dimensions relatively accurately. The egocentric VR perspective substantially modifies the way users see specific body parts, such as hips, thorax, and waist, which people from both general and clinical populations seem to overestimate within the context of body size dissatisfaction [50,51]. Finally, egocentric VR perspective exploration tended to show an experience based on intra-individual comparison to one’s own body (particularly for body distortion) rather than inter-individual comparison common within an allocentric perspective.

### 4.3. Believing and Feeling: Source of Discrepancy

Outcomes from two psychometric and exploratory objectives (O.1 and O.2) suggested that the egocentric perspective measured a different and possibly complementary dimension of BIDs compared to the allocentric perspective (from both traditional paper-based FRS and VR), as proposed in previous studies [29,42]. The difference in BID ratings may be due to variation in how participants use their abstract knowledge, beliefs, and feelings about their bodies within the two perspectives. In BIDs, beliefs and behaviors related to one’s own body are informed by an allocentric perspective, namely, the idea of “what people think they have to look like”. Since real-time perception driven inputs inform the egocentric perspective, we can hypothesize that the egocentric perspective could primarily reflects the perceptual-affective construction of BIDs, with perceptual body distortion deriving mainly from “intra-individual comparisons”. In contrast, we can hypothesize that, since the allocentric perspective reflects the cognitive-affective construction of BIDs, body dissatisfaction would be deriving mainly from “inter-individual comparisons”. This contrast echoes Cash’s trait- and state-based theory [10], which indicates that BIDs function based on both “trait BIDs” from inter-individual variability due to socio-contextual cues (pair comparison, bodily remarks, etc.) and “state BIDs” from intra-individual variability due to personal cues (mood, bodily sensation, etc.). State BIDs are thought to fluctuate on a moment-by-moment basis, and these fluctuations are mainly associated with current mood and individual differences in personality dispositions and disordered eating symptomatology [52,53,54]. In line with Cash’s theory (demonstrated empirically by Colautti et al. [54]), an egocentric perspective integrates the internal stimuli that have been described as the subjective felt experience of the body, called “embodiment” [55,56]. In a VR task, embodiment refers to the simulation of a virtual body to create new spatial perceptions. The embodied cognitive theory claims that our inner body experience (feeling) is shaped by our mental representation of the body. In other words, this theory posits that individuals could actually feel the body that they think they have. Recent studies have indicated that VR enhances the sense of embodiment, influences the body schema, and modulates action performance [57], and especially when the virtual and real images are close [58]. This specific effect of VR could explain a part of our results and should be explored in future studies by comparing 3D avatars to the same avatars on a 2D screen. The Rubber Hand Illusion is an example of the cognitive process of “feeling” a limb by making our brain “believe” that our body actually carries this rubber hand as a real limb. Even if participants do not believe the rubber hand is part of their own body, they may feel that it is. Recently, Tamé, Linkenauger, and Longo [59] demonstrated that, in the Rubber Hand Illusion task, the sense of body ownership was significantly stronger when participants were asked to judge their feelings rather than beliefs about the hand. In the same way, the assessment tools, and therefore the phrasing of items, were first developed for assessing the external body representation. They may refer more to beliefs (external aspect and cognitive construct), instead of feelings (sensation, emotion, and body internalization). It supports the notion of referring to the appraisal and rating of 3D virtual bodies instead of just 2D figures. Finally, this new hypothesis conceives egocentric and allocentric perspectives on BDIs as a double-sided coin, with “egocentric perceptual-affective-attitudinal BIDs” on one side and “allocentric perceptual-affective-attitudinal BIDs” on the other side, unlike the complex somatoperception-based lock theory [38].

### 4.4. Implications For Intervention

The contribution of the allocentric VR perspective may be promising in studying self-objectification (i.e., the tendency to experience the body from an external-observer point of view) and appearance-ideal internalization in order to prevent EDs, particularly in young female students [4,60]. This implication is in line with recent findings on the advantages of using VR in school [61] to promote integrated prevention programs focused on physical self-perceptions [62]. Although the study was conducted within the general population, the hypothesized proportion of the ED population within the study sample could be extrapolated to explore potential implications for the clinical population. It could be interesting to investigate the use of the egocentric VR perspective in assessing the awareness of bodily sensation that is normally a deficit in the clinical anorexic population [63]. On that note, it is worth mentioning that study participants anecdotally noted that immersions in VR made them more aware of their body size distortion and their difficulty in defining what would be an appropriate silhouette/virtual body for them. This raises the possibility of considering interventions that target individuals’ feelings during the immersions to improve their ability to be aware of bodily feelings. This point refers to a recent nutritional approach in ED treatment—intuitive eating—which emphasizes respect for the body, regardless of weight and shape, and encourages the use of mindfulness techniques to focus on physical cues for hunger and satiety [64]. Exploring these bodily sensations from an egocentric perspective could make it easier to reconnect with one’s own food-related sensations.

### 4.5. Strengths and Limitations

To our knowledge, one significant contribution of the current study is to innovate by assessing BIDs from allocentric and egocentric VR perspectives, thus validating *e*LoriCorps within the general population. For example, in Fisher et al.’s study [29], their scores were correlated to validated psychometric questionnaire data on BIDs, but the task did not allow researchers to document the convergent and discriminant validity of the tool. Beyond convergent and discriminant validity, the VR perspectives of *e*LoriCorps could improve participants’ motivation to measure their body image, thanks to the ability to virtually walk around silhouettes and see bodies from different perspectives. Integrating allocentric and egocentric perspectives in its development, *e*LoriCorps allowed the researcher to assess cognitive-affective and perceptual dimensions related to one’s own body to determine BIDs. Methodological strengths were also worth noting. The sample size was relatively large compared to sample sizes in other studies [29,44]. The current study presented some limitations, which were related to the psychometric instruments and the position of the virtual bodies. The first main limitation was the lack of “gold-standard” psychometric instruments to compare with the egocentric VR perspective, which is a novel tool. Moreover, beyond allocentric and egocentric perspectives, convergent/discriminant validity underlined that the methods used to evaluate body distortion—based on a single perception (actual body size)—and body dissatisfaction—based on two perceptions (actual and desired body size)—impacted the effect sizes of correlation. Secondly, in the egocentric perspective, the virtual bodies were not presented in a completely frontal position, providing less capability for participants to see the bodies in their entirety. It could be interesting to recreate an egocentric virtual body rating scale in which participants could lean forward to see every part of their body. Another alternative that must be studied further is that what is actually viewed by the participants in the allocentric and egocentric perspectives differs also in terms of body parts, of potential aesthetics qualities, and brain mechanisms, allowing the identification of body parts. Carey et al. [65] studied the role of a cortical region dedicated to the visual processing of bodies and body parts. Their results suggested that the extrastriata body area was activated differentially in the allocentric and egocentric perspectives. Their findings might not apply to our non-clinical sample, as they suggested that alteration in the functioning of this brain area was a consequence of eating disorders psychopathology, but this is an area deserving more research attention. Finally, when considering the findings of our study, it is important to mention we did not control for the presence of eating disorders or other psychiatric comorbidities in the participants. Further studies should include specific ED populations. In addition, this study was conducted with a rather young sample. The impact of potential social-cultural factors related to body image was not measured and controlled (e.g., frequency of exposure to social-medial content). Further studies should explore if the results are replicated in older samples or influences by social-cultural factors. Of note, it is also important to study the test-retest reliability of the *e*LoriCorps in the future.

## 5. Conclusions

In conclusion, our results provide evidence that the allocentric version of *e*LoriCorps is a valid tool to assess a range of BIDs, including cognitive-affective and perceptual dimensions. Moreover, our study highlights the potential of investigating BIDs through both allocentric and egocentric perspectives. Indeed, we propose that both perspectives may be complementary: egocentric VR perspective exploration tend to provide an experience based on intra-individual comparison to one’s own body (particularly for body distortion) rather than inter-individual comparison, which could occur with an allocentric perspective. Moreover, the contribution of the egocentric perspective to measure and possibly treat BIDs in general and clinical populations is promising and must be explored in future studies. Because binge eating disorder was added to the last Diagnostic and Statistical Manual of Mental Disorders [6], *e*LoriCorps 2.0 should present a wider range of virtual bodies to represent the diversity of existing bodies and morbid obesity forms that may be related to this eating disorder. Besides, it could be interesting to develop a specific adolescent virtual bodies continuum to have a more authentic representation of the bodies of this at-risk population for EDs.

## Figures and Tables

**Table 1:** Descriptive statistics of actual BMI and ratings of body image based on a classical paper-based FRS or *e*Loricorps allocentric and egocentric VR conditions.

	M	SD	Min–Max
Actual BMI ^1^	23.24	4.84	16.7–47.6
Perceived body size score			
Paper-based FRS ^2^	3.92	0.97	2–6
Allo. VR ^3^	3.80	0.81	2–6
Ego. VR ^4^	3.48	1.41	1–6
Ideal body size score			
Paper-based FRS	3.22	0.60	2–4
Allo. VR	3.20	0.64	1–4
Ego. VR	3.56	1.29	1–7
Body dissatisfaction score			
Paper-based FRS	0.91	0.65	0–3
Allo. VR	0.61	1.01	−2–4
Ego. VR	−0.08	1.23	−3–3
Body distortion score			
Paper-based FRS	0.12	1.02	−2–4
Allo. VR	0.25	1.10	−2–4
Ego. VR	0.57	1.59	−3–6

^1^ body mass index, ^2^ figure rating scale, ^3^ allocentric virtual reality condition, ^4^ egocentric virtual reality condition.

**Table 2:** Spearman correlations between traditional paper-based FRS mode and allocentric and egocentric VR conditions for each component of body image disturbances (BIDs).

	Spearman Rho	*p*-Value
Perceived Body Size Score		
Paper-based FRS ^1^ vs. Allo. VR ^2^	0.744	<0
Paper- based FRS vs. Ego. VR ^3^	0.459	<0
Allo. VR vs. Ego. VR	0.440	<0
Ideal Body Size Score		
Paper-based FRS vs. Allo. VR	0.195	0.116
Paper- based FRS vs. Ego. VR	0.201	0.106
Allo. VR vs. Ego. VR	0.109	0.390
Body Distortion Score		
Paper-based FRS vs. Allo. VR	0.783	<0
Paper- based FRS vs. Ego. VR	0.487	<0
Allo. VR vs. Ego. VR	0.479	<0
Body Dissatisfaction Score		
Paper-based FRS vs. Allo. VR	0.472	<0
Paper- based FRS vs. Ego. VR	0.160	0.198
Allo. VR vs. Ego. VR	0.282	0.022

^1^ figure rating scale, ^2^ allocentric virtual reality condition, ^3^ egocentric virtual reality condition.

**Table 3:** Correlation between egocentric VR score (compared to allocentric VR score) and external variables for BIDs’ components.

	Drive for Thinness Subscale (EDI ^1^)	Bulimia Subscale (EDI)	Body Dissatisfaction Subscale (EDI)	Symptoms Subscale (EDI)	SPAS12 ^2^
Body Distortion Score					
Allo. VR ^3^	−0.453 (0.004)	−0.394 (0.003)	−0.206 (0.120)	−0.471 (>0)	−0.339 (0.006)
Ego. VR ^4^	−0.280 (0.035)	−0.218 (0.105)	0.015 (0.913)	−0.234 (0.079)	−0.326 (0.008)
Body Dissatisfaction Score					
Allo. VR	−0.185 (0.165)	−0.126 (0.345)	−0.175 (0.186)	−0.141 (0.290)	0.489 (<0)
Ego. VR	−0.092 (0.495)	−0.166 (0.214)	0.047 (0.726)	−0.164 (0.219)	0.281 (0.023)

^1^ eating disorder inventory-2, ^2^ social and physical anxiety scale, ^3^ allocentric virtual reality condition, ^4^ egocentric virtual reality condition.
